# The Effect of Neddylation Inhibition on Inflammation-Induced MMP9 Gene Expression in Esophageal Squamous Cell Carcinoma

**DOI:** 10.3390/ijms22041716

**Published:** 2021-02-09

**Authors:** Anita Hryniewicz-Jankowska, Jaroslaw Wierzbicki, Renata Tabola, Kamilla Stach, Khalid Sossey-Alaoui, Katarzyna Augoff

**Affiliations:** 1 Laboratory of Cytobiochemistry, Biotechnology Faculty, University of Wroclaw, 50-383 Wroclaw, Poland; anita.hryniewicz-jankowska@uwr.edu.pl; 2 Department of Minimally Invasive Surgery and Proctology, Wroclaw Medical University, 50-556 Wroclaw, Poland; jaroslaw.wierzbicki@umed.wroc.pl; 3 Second Department and Clinic of General and Oncological Surgery, Wroclaw Medical University, 50-556 Wroclaw, Poland; renata.tabola@umed.wroc.pl; 4 Department of Biochemistry, Wroclaw Medical University, 50-368 Wroclaw, Poland; kamilla.stach@umed.wroc.pl; 5 Department of Medicine, School of Medicine, Case Western Reserve University, Rammelkamp Center for Research, Cleveland, OH 44109, USA; kxs586@case.edu; 6 Department of Surgical Education, Wroclaw Medical University, 50-368 Wroclaw, Poland

**Keywords:** neddylation, metalloproteinases, cancer cell migration, TNF-α–NFκB signaling pathway, esophageal squamous cell carcinoma

## Abstract

Inhibition of the protein neddylation process by the small-molecule inhibitor MLN4924 has been recently indicated as a promising direction for cancer treatment. However, the knowledge of all biological consequences of MLN4924 for cancer cells is still incomplete. Here, we report that MLN4924 inhibits tumor necrosis factor-alpha (TNF-α)-induced matrix metalloproteinase 9 (MMP9)-driven cell migration. Using real-time polymerase chain reaction (PCR) and gelatin zymography, we found that MLN4924 inhibited expression and activity of MMP9 at the messenger RNA (mRNA) and protein levels in both resting cells and cells stimulated with TNF-α, and this inhibition was closely related to impaired cell migration. We also revealed that MLN4924, similar to TNF-α, induced phosphorylation of inhibitor of nuclear factor kappa B-alpha (IκB-α). However, contrary to TNF-α, MLN4924 did not induce IκB-α degradation in treated cells. In coimmunoprecipitation experiments, nuclear IκB-α which formed complexes with nuclear factor kappa B p65 subunit (NFκB/p65) was found to be highly phosphorylated at Ser32 in the cells treated with MLN4924, but not in the cells treated with TNF-α alone. Moreover, in the presence of MLN4924, nuclear NFκB/p65 complexes were found to be enriched in c-Jun and cyclin dependent kinase inhibitor 1 A (CDKN1A/p21) proteins. In these cells, NFκB/p65 was unable to bind to the MMP9 gene promoter, which was confirmed by the chromatin immunoprecipitation (ChIP) assay. Taken together, our findings identified MLN4924 as a suppressor of TNF-α-induced MMP9-driven cell migration in esophageal squamous cell carcinoma (ESCC), likely acting by affecting the nuclear ubiquitin–proteasome system that governs NFκB/p65 complex formation and its DNA binding activity in regard to the MMP9 promoter, suggesting that inhibition of neddylation might be a new therapeutic strategy to prevent invasion/metastasis in ESCC patients.

## 1. Introduction

Esophageal squamous cell carcinoma (ESCC) is a predominant histological type accounting globally for about 87% of all esophageal cancers ranked as the sixth leading cause of cancer death worldwide [1]. While ESCC incidence may vary according to geographic region, it comprises about 1% of all cancers diagnosed each year [2]. ESCC is known as one of the most aggressive, fast growing and metastasizing cancers. Despite recent improvements in diagnostics and therapy with neoadjuvant chemotherapy or radiochemotherapy, the 5-year survival rate for ESCC patients is low and does not exceed 20% [2].

The major risk factors established for ESCC are tobacco smoking and alcohol consumption [1,3,4]. Recurrent long-term exposure of esophageal epithelium to harmful factors is known to cause tissue damage and chronic inflammation which leads to constitutive activation of signaling pathways, promoting cell survival and proliferation [5,6]. Recently, chronic inflammation has been indicated as an additional contributor to ESCC pathogenesis [7]. Several pro-inflammatory cytokines, such as tumor necrosis factor-alpha (TNF-α), secreted mostly by cells of the immune system, have been shown to be upregulated in patients with ESCC, indicating their potential effect on the tumor development [7,8].

TNF-α is a well-known activator of pro-invasive and pro-migratory genes including matrix metalloproteinase 9 (MMP9/gelatinase B) by activation of the transcription factors such as the nuclear factor kappa-light-chain-enhancer of activated B cells (NFκB) or the activator protein 1 (AP-1) [9,10,11]. MMP9 is a major mediator of the degradation of extracellular matrix (ECM) proteins such as collagen, fibronectin and laminin, and its activation is a key event in the ECM remodeling process closely associated with cancer cell migration [10,12]. In addition to regulating ECM organization, MMP9 degradation of non-matrix substrates such as chemokines, growth factors or integrins contributes to the control of signals promoting migration of both tumor and stromal cells [13,14]. According to the literature, the expression and the activity of various matrix metalloproteinases (MMPs), including MMP9, were found to be upregulated in more than 60% of esophageal squamous cell carcinoma cases [13,15]. Our previous study showed that impaired activity of not only gelatinases, but also of serine proteases such as fibroblast activation protein alpha (FAPα) and dipeptidyl peptidase IV (DPPIV/CD26) is closely associated with ESCCs [16]. In our recent work, we demonstrated that MMP9 is directly involved in TNF-α-induced breast cancer cell migration [17].

It is obvious that NFκB is an essential transcription factor for TNF-α-dependent MMP9 expression and the inhibition of NFκB activity significantly reduces MMP9 secretion [18,19,20]. In light of recent research, cullin-RING (really interesting new gene) ligase (CRL) neddylation was indicated as the relevant step in control of ubiquitin-dependent proteasomal degradation of nuclear factor kappa B (IκB) proteins, inhibitory molecules conditioning NFκB activity. Neddylation is one of the common posttranslational modifications (PTMs) of proteins by covalent attachment of a ubiquitin-like protein NEDD8 (neural precursor cell-expressed developmentally downregulated protein 8) to a lysine residue of target proteins mediated by an E1 NEDD8-activating enzyme (NAE), an E2 NEDD8-conjugating enzyme and an E3 NEDD8 ligase in sequential steps [21]. The binding of NEDD8 induces a conformational change of the cullin C-terminal domain, and as a result, it enhances Ctrl ubiquitination activity [22]. Although the cullin family members are the best-known and studied neddylation targets, other non-cullin proteins including various transcription factors such as tumor protein 53 (TP53), tumor protein 73 (TP73) and E2F have been recently shown to be tagged with NEDD8 and dysregulation of the neddylation appears to be one of the hallmarks of cancers [23].

A decade ago, a highly selective small-molecule inhibitor of NAE, MLN4924 (also known as pevonedistat), was developed to interrupt the growth of tumor cells through the inhibition of CRL-dependent protein degradation, resulting in accumulation of intracellular suppressors of signaling pathways [24]. Because MLN4924 could affect the cell cycle, DNA replication, DNA damage response and cell motility, it has been recognized as a promising anticancer agent, and is undergoing phase I or phase II clinical testing for several hematological and solid tumors [25,26,27]. Although in many studies MLN4924 was shown to indirectly block NFκB activation, the exact mechanism of action of MLN4924 in the regulation of NFκB-dependent gene expression is still not fully elucidated.

The aim of this study was to investigate the effects of MLN4924 on TNF-α-induced expression and secretion of MMP9 in human esophageal squamous cancer cells and to shed some light on the mechanism underlying these processes. We revealed that MLN4924 totally blocks interactions between NFκB and the MMP9 gene promoter likely by disruption of the stoichiometric balance within components of the NFκB complex. In this study, we demonstrated for the first time that three of CRL’s substrates, inhibitor of nuclear factor kappa B (IκB), c-Jun and cyclin dependent kinase inhibitor 1 A (CDKN1A/p21), are potentially responsible for the loss of functionality of the NFκB complex in the nucleus. Our results suggest an important role of neddylation inhibition in the dysregulation of nuclear ubiquitin–proteasome system (UPS), in particular when it comes to anticancer therapy.

## 2. Results

### 2.1. MLN4924 Downregulates TNF-α-Induced MMP9 Expression in ESCC Cells

**Figure 1:**
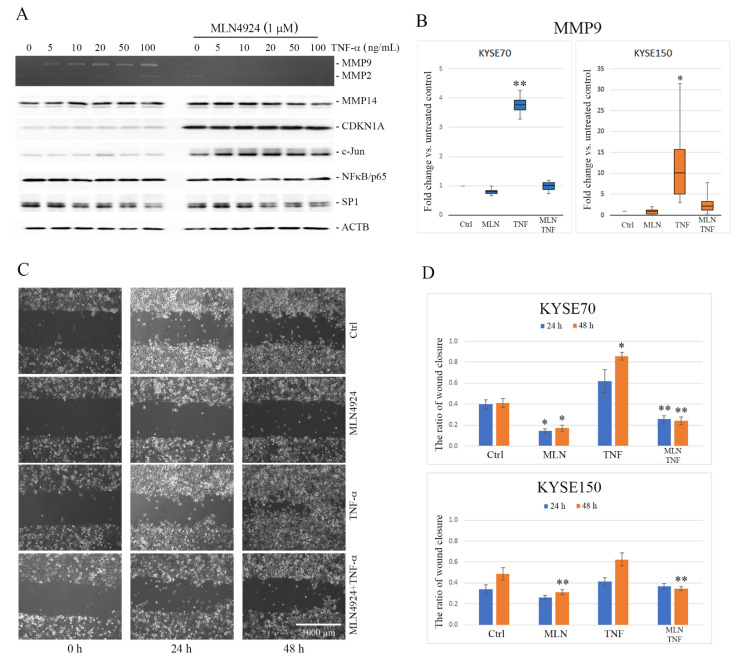
Effect of tumor necrosis factor-alpha (TNF-α) and MLN4924 on matrix metalloproteinase 9 (MMP9) expression and esophageal squamous cell carcinoma (ESCC) cell migration. (**A**) After overnight serum starvation, KYSE150 cells were pretreated or not with 1 μM MLN4924 for 30 min and then stimulated with TNF-α at the indicated concentration for 24 h. Activity of MMP9 and MMP2 was analyzed by gelatin zymography in the conditioned media. The protein levels of membrane type I-matrix metalloproteinase (MT1-MMP), cyclin dependent kinase inhibitor 1A (CDKN1A/p21), c-Jun, nuclear factor kappa B (NFκB) and SP1 were determined by Western blotting. Fold change calculated as the ratio of relative levels of proteins normalized to β-actin (ACTB) between the cells treated with TNF-α in combination with MLN4924 and the cells treated with TNF-α alone is shown in Figure S4. Values shown are means ± SEM: whiskers: min–max. (**B**) MMP9 messenger RNA (mRNA) expression was analyzed by reverse transcription quantitative real-time polymerase chain reaction (RT-qPCR) in both KYSE150 and KYSE70 cells treated for 24 h with TNF-α (30 ng/mL) and MLN4924 (1 µM) alone or in combination. Results are presented as fold change of *MMP9* gene in the treated cells relative to the untreated controls normalized to the expression of the reference gene encoding glyceraldehyde 3-phosphate dehydrogenase (*GAPDH*). (**C**) Representative images from wound healing assay showing the changes in KYSE70 cell migration under MLN4924 and TNF-α applied separately and in combination after 24 h and 48 h treatment in relation to the untreated controls. The cells were seeded in silicone inserts and allowed to attach and form a confluent monolayer. After the inserts were removed, the cells were treated with TNF-α (30 ng/mL) and MLN4924 (1 µM) separately or in combination and incubated for the next 48 h. Images of cell-free artificial wounds were taken at 0, 24 and 48 h. (**D**) Quantitative analysis of wound closure. Summary bar graphs illustrating the ratio of wound closure after 24 and 48 h quantified for KYSE150 and KYSE70 cells. Values shown are means ± SEM. * *p* < 0.001, ** *p* < 0.05 vs. control. Ctrl—untreated controls.

**Figure 2:**
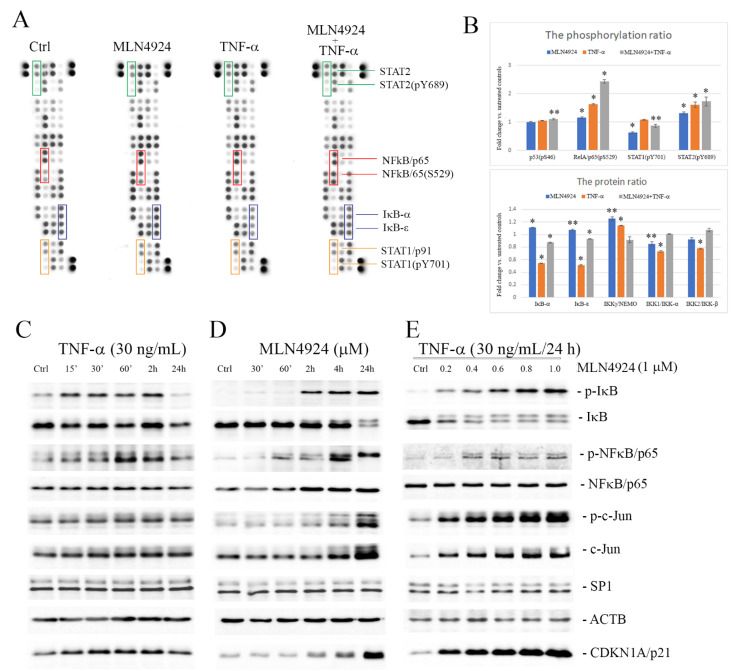
Effect of tumor necrosis factor-alpha (TNF-α) and MLN4924 on the signaling pathways mediating matrix metalloproteinase 9 (MMP9) gene expression in esophageal squamous cell carcinoma (ESCC) cells. (**A**) Proteome profiling of the nuclear factor kappa B (NFκB) pathway by antibody array analyses in the KYSE150 cells treated with MLN4924 and TNF-α. Protein lysates from the untreated controls and cells treated with MLN4924 or TNF-α alone or in combination were analyzed using a human NFκB array (R&D). (**B**) Bar graphs showing the phosphorylation ratio (upper) and the protein level ratio (bottom) calculated after the semi-quantitative analysis of selected proteins. Results are presented as means ± SEM from duplicates. * *p* < 0.001, ** *p* < 0.05 vs. controls. The complete array is shown in Figure S3. (**C**) Western blot analysis showing time-dependent activation of inhibitor of nuclear factor kappa B-alpha (IκB-α), NFκB/p65 and c-Jun in the KYSE150 cells treated with TNF-α (30 ng/mL). (**D**) MLN4924-dependent changes in the activation of IκB-α, NFκB/p65 and c-Jun as well as increasing levels of cyclin dependent kinase inhibitor 1A (CDKN1a/p21) protein in the KYSE150 cells within 24 h. (**E**) A dose-dependent effect of MLN4924 on activation of NFκB/p65 and c-Jun signaling pathways in the KYSE150 cells treated with TNF-α (30 g/mL) for 24 h. The effect of the KYSE70 cells treatment with different concentrations (0.25, 0.5, 1.0, 2.5 and 5.0 µM) of MLN4924 for 24 and 48 h is shown in Figure S2.

We observed by gelatin zymography or Western blotting that the treatment of ESCC cells with TNF-α significantly increased secretion of MMP9, but not of MMP2 or MMP14 (also known as membrane type 1-matrix metalloproteinase, MT1-MMP), in a dose-dependent manner (Figure 1A and Figure S1). Upregulation of MMP9 gene expression induced by TNF-α was confirmed by the quantitative real-time polymerase chain reaction (qPCR) assay and showed nearly 4-fold and 14-fold increase in the MMP9 mRNA level in KYSE70 and KYSE150 cells, respectively, under TNF-α (Figure 1B). The pre-treatment of ESCC cells with MLN4924 inhibited TNF-α-induced secretion of MMP9 in both KYSE70 and KYSE150 cells. In gelatin zymograms, activity of MMP9 in the conditioned medium was undetectable even at MLN4924 concentration as low as 0.2 µM. The inhibitory effect of MLN4924 on MMP9 expression was verified by reverse transcription quantitative real-time polymerase chain reaction (RT-qPCR). As shown in Figure 1B, in the cells treated with MLN4924 alone, a meaningful reduction of the MMP9 mRNA level (fold change = 0.7 and 0.4 in KYSE70 and KYSE150, respectively) to below the basal level found in control untreated cells (*p* < 0.05) was observed. In the cells treated with TNF-α combined with MLN4924, the mRNA level of MMP9 was similar to that observed in untreated cells and remarkably lower as compared with the cells stimulated with TNF-α alone. The differences were statistically significant (*p* = 0.01). To determine the role of MLN4924 in modulating the availability of transcriptional regulators of the *MMP9* gene promoter, the levels of NFκB/p65, SP1, c-Jun and CDKN1A/p21 proteins were analyzed by Western blotting in KYSE150 cells. We observed a significant increase in levels of c-Jun and CDKN1A/p21 in a dose- and time-dependent manner in the cells treated with MLN4924 as compared with non-stimulated cells or those stimulated with TNF-α alone (Figure 1A, Figure 2 and Figure S1).

These data provide evidence that MLN4924 selectively inhibits MMP9 activity in ESCC cells and confirm the presence of alterations in the composition of MMP9-associated transcriptional regulators under the treatment with MLN4924.

### 2.2. MLN4924 Inhibits Migration of ESCC Cells *In Vitro*

Our previous studies have shown that TNF-α significantly enhanced the migratory and invasive potentials of breast cancer cells [15]. In this study, using the wound healing assay, we observed that TNF-α boosted ESCC cell motility (Figure 1C,D). The differences between the untreated cells and cells treated with TNF-α were found to be statistically significant for KYSE70 cells after incubation for 24 h (*p* < 0.0005) and 48 h for both KYSE70 and KYSE150 cells (*p* < 0.0005 and *p* < 0.01, respectively). In the presence of MLN4924, the pro-migratory activity of TNF-α on esophageal squamous cancer cells was significantly reduced (*p* < 0.005). Motility of the cells treated with TNF-α in combination with MLN4924 was significantly lower even when compared with untreated control cells (*p* < 0.05). The ratio of artificial wound closure in the culture treated with MLN4924 only was found to be the lowest of all and this effect was statistically significant at both 24 h and 48 h as compared with other cells (Figure 1D). As we observed, the treatment of ESCC cells with MLN4924 with a range of concentrations up to 1 μM did not result in changes in cell viability within 24 h. Cell growth decreased by 50% in both KYSE70 and KYSE150 after 48 h of treatment with MLN4924, but not with TNF-α (Figure S2). However, only the treatment of ESCC cells with MLN4924 at the concentration ≥ 25 μM led to cell apoptosis confirmed by alterations in cell morphology and cleavage of PARP1 (proteins including poly(ADP-ribose) polymerase-1) in an 89 kDa fragment (Figure S3).

These data confirm that MLN4924 may inhibit migration of ESCC cells independently of apoptosis.

### 2.3. MLN4924 Upregulates Phosphorylation of IκB-α and NFκB/p65

The role of neddylation in the regulation of NFκB signaling is well-known. In this study, we used a membrane-based antibody array to evaluate relative levels of 45 selected NFκB pathway proteins in the cells exposed to MLN4924 or TNF-α (Figure S4). The level of phosphorylation of p53 (Ser46), NFκB/p65 (Ser529), STAT1 (Tyr701) and STAT2 (Tyr689) was determined and normalized to total proteins (Figure 2A). The treatment with MLN4924 and TNF-α, either separately or in combination, resulted in a 1.1-fold, 1.6-fold and 2.4-fold increase, respectively, in NFκB/p65 (Ser529) phosphorylation and a 1.3-fold, 1.6-fold and 1.7-fold increase, respectively, in STAT2 (Tyr689) phosphorylation as compared with the untreated controls. On the other hand, the phosphorylation level of STAT1 (Tyr701) was found to be decreased with a fold change = 0.6 under MLN4924 treatment. Changes in the phosphorylation levels of p53 (Ser46) were not observed. Of the other proteins, NFκB inhibitors, IκB-α and IκB-ε, and two IκB kinases, IKK1 and IKK2, were downregulated in the TNF-α-stimulated cells with a fold change = 0.5 and 0.7, respectively, but they remained unchanged in the presence of MLN4924 when compared with untreated control cells (Figure 2B).

Thereafter, to clarify the mechanism by which NFκB/p65 may be involved in MLN4924-dependent inhibition of TNF-α-induced MMP9 gene expression in ESCC cells, the levels of phospho-NFκB/p65 (Ser536) and phospho-IкB-α (Ser32) were analyzed in whole cell lysates in the time–course and dose–response experiments by Western blotting (Figure 2C–E and Figure S5). As was expected, the phosphorylation levels of IκB-α and NFκB/p65 increased transiently upon TNF-α treatment. Minor degradation of IκB-α also occurred and a progressive decrease in total IκB-α level was seen with time (Figure 2C). Unlike the classical IκB-α phosphorylation/degradation process induced by TNF-α, the MLN4924-related phosphorylation of IκB-α at Ser32 occurred over one hour and increased in a time-dependent manner (Figure 2D). It was closely correlated with lowered levels of IκBα in its 39-kDa form and with the presence of an additional band (~45 kDa), which most likely resulted from the impaired degradation of highly phosphorylated IκB-α.

In a similar way, the treatment of cells with MLN4924 upregulated levels of phosphorylated NFκB/p65 in a time- and dose-dependent manner (Figure 2D,E), reaching a plateau at the MLN4924 concentration of 2.5 μM after 48 h of incubation. The increase in total c-Jun under MLN4924 was found to be positively correlated with the phosphorylation level at Ser73. Treatment of ESCC cells with varying concentrations of MLN4924 up to 5.0 µM within 48 h did not lead to changes in total NFκB/p65 and SP1 levels (Figure S5).

These data indicate that MLN4924 by itself upregulates phosphorylation of both IκB-α and NFκB/p65, as well as c-Jun, in a time- and dose-dependent manner in ESCC cells, suggesting its ability to activate NFκB signaling.

### 2.4. MLN4924 Dysregulates Subcellular Localization of Phospho-NFκB/p65 and Phospho-IκB-α

**Figure 3:**
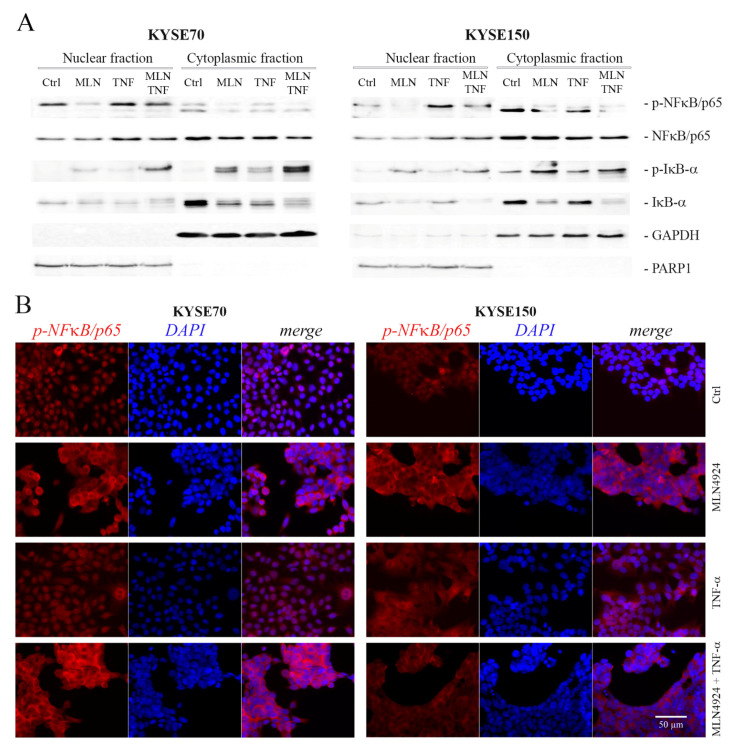
Subcellular localization of phospho-inhibitor of nuclear factor kappa B-alpha (phospho-IκB-α) and phospho- nuclear factor kappa B p65 subunit (phospho-NFκB/p65) in KYSE70 and KYSE150 cells under treatment with tumor necrosis factor-alpha (TNF-α) and MLN4924. (**A**) Western blot analysis of changes in cellular localization of phospho-IκB-α (S32) and phospho-NFκB/p65 (S536) in response to 24 h treatment with TNF-α (30 ng/mL) and MLN4924 (1 µM) applied separately or in combination. Protein cell lysates were separated into cytoplasmic and nuclear fractions which were probed for both total and phosphorylated forms of IκB-α and NFκB/p65. Glyceraldehyde 3-phosphate dehydrogenase (GAPDH)—cytosolic marker; Poly (ADP-ribose) polymerase 1 (PARP1)—nuclear marker. (**B**) Immunofluorescence staining showing subcellular localization of phospho-NFκB /p65 (S536). Untreated KYSE150 and KYSE70 cells (Ctrl) or cells treated with MLN4924 (1 µM/24 h) or TNF-α (30 ng/mL/1 h) separately or in combination were fixed and probed against NFκB/p65 phosphorylated at Ser536 (shown in red). The cell nuclei were additionally counterstained with DAPI (shown in blue). Unstimulated cells showed a weak signal from phospho-NFkB/p65, localized both to the cytoplasm and the nucleus. In the cells treated with TNF-α, phospho-NFκB/p65 localized mostly to the nucleus. Phospho-NFκB/p65 was predominantly localized to the cytoplasm in the presence of MLN4924.

To analyze the effects of MLN4924 and TNF-α on intracellular localization of phosphorylated forms of NFκB/p65 (Ser536) and IκB-α (Ser32), cytoplasmic and nuclear fractions were separated. In Western blotting, positive signals for phospho-NFκB/p65 were observed in nuclear cell fractions of all cells, both untreated and treated. In the presence of MLN4924, the level of nuclear phospho-NFκB/p65 was slightly lowered as compared with that of control untreated cells. However, a significantly increased phospho-NFκB/p65 level was found in nuclei of the cells treated with TNF-α alone, as well as in combination with MLN4924. Contrary to NFκB/p65, the levels of nuclear phospho-IκB-α were found to be remarkably upregulated in the cells treated with MLN4924 but only slightly increased in the cells treated with TNF-α only as compared to untreated controls. A similar relationship was observed in cytoplasmic cell fractions. Interestingly, we found dramatically lowered levels of total IκB-α in the cells treated with both MLN4924 and TNF-α in relation to untreated controls (Figure 3A).

The changes in the localization of phosphorylated NFκB/p65 at Ser536 in ESCC cells treated with MLN4924 and TNF-α applied separately or in combination were also investigated by immunocytochemistry. In the presence of MLN4924, phospho-NFκB/p65 was predominantly localized in the cytoplasm. However, the phospho-NFκB/p65-related fluorescence could also be observed in nuclei of many MLN4924-treated cells. Contrary to MLN4924, phospho-NFκB/p65 in response to TNF-α localized mostly to the nucleus (Figure 3B).

Taken together, our findings show that MLN4924 treatment significantly impairs the passage of phosphorylated forms of NFκB/p65 from the cytosol into the nucleus and simultaneously increases nuclear levels of phospho-IκB-α.

### 2.5. MLN4924 Generates Nuclear NFκB/p65-IκB-α Complexes Rich in Phospho-IκB-α (Ser32) and c-Jun/phospho-c-Jun (Ser73)

**Figure 4:**
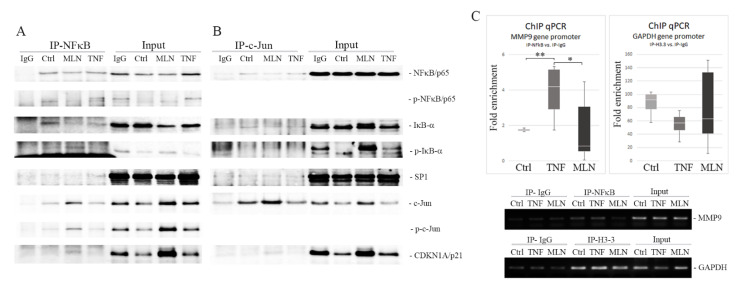
Western blot analysis of the nuclear factor kappa B (NFκB) complex and the chromatin immunoprecipitation (ChIP)-quantitative polymerase chain reaction (qPCR) assay of the matrix metalloproteinase 9 (*MMP9*) gene promoter in KYSE150 cells. Nuclear protein-enriched extracts from esophageal squamous cell carcinoma (ESCC) cells were used for co-immunoprecipitation (co-IP) with a mouse anti-NFκB/p65 antibody (**A**) or with a rabbit anti-c-Jun antibody (**B**) and probed for inhibitor of nuclear factor kappa B-alpha (IκB-α)/phospho-IκB-α, SP1 and cyclin dependent kinase inhibitor 1A (CDKN1A/p21) as well as phosphorylated and unphosphorylated forms of NFκB/p65 and c-Jun. Immunoglobulin G (IgG) mixture (1:1:1) of each protein extract and normal mouse IgG or normal rabbit IgG, respectively, used as negative controls; Ctrl—protein extract from untreated cells; MLN4924 and tumor necrosis factor-alpha (TNF-α)—extracts from cells treated with MLN4924 (1 µM) for 24 h and with TNF-α (30 ng/mL) for 1 h, respectively. (**C**) ChIP-qPCR assay on the *MMP9* gene promoter in KYSE150 cells. Sheared chromatin was immunoprecipitated with antibodies against NFκB/p65 and with an anti-acetyl histone H3.3 antibody, used as a positive control. Normal rabbit IgG served as a negative antibody control. Box plots showing qPCR data normalized with the fold enrichment method. Values shown are means ± SEM: whiskers: min–max; * *p* < 0.001, ** *p* < 0.01 vs. controls. Agarose gels showing DNA fragments amplified with MMP9 and glyceraldehyde 3-phosphate dehydrogenase (GAPDH) primers.

To investigate the ability of NFκB/p65 to form nuclear complexes with its protein partners, coimmunoprecipitation studies were performed on the nuclear fractions of cells treated with MLN4924 or TNF-α using the anti-NFκB/p65 antibody. Using Western blot analysis, we found that nuclear NFκB/p65 coimmunoprecipitated with both IκB-α and transcription factors such as SP1 and c-Jun in both treated and untreated cells (Figure 4A). However, the presence of MLN4924 resulted in a considerably increased signal of phospho-IκB-α (Ser32) as well as of c-Jun/phospho-c-Jun (Ser73) with NFκB when compared with the cells treated with TNF-α alone or untreated controls. Moreover, we revealed that CDKN1A/p21, which is known to regulate TNF-α-induced MMP9 gene expression, was also coprecipitated with NFκB/p65 in the cells treated with MLN4924. Coimmunoprecipitation analysis performed with the use of an antibody directed against c-Jun confirmed the presence of IκB in nuclear complexes formed between NFκB and c-Jun in both resting and stimulated cells (Figure 4B).

These data confirm that the composition of NFκB nuclear complexes is significantly impaired in the presence of MLN4924, resulting in excessive levels of phosphorylated forms of IκB-α and c-Jun. Additionally, CDKN1A/p21 protein was identified in NFκB/p65-containing complexes in the cells treated with MLN4924. Therefore, the role of CDKN1A/p21 seems to be important, too, in this arrangement.

### 2.6. MLN4924 Blocks NFκB/p65–DNA Binding

To determine the inhibitory effect of MLN4924 on the binding ability of NFκB to the MMP9 promoter, the chromatin immunoprecipitation quantitative real-time PCR (ChIP-qPCR) assay was performed using an antibody directed against NFκB. Histone H3.3 antibody and normal rabbit IgG served as the positive and negative controls, respectively, in this assay. Contrary to non-treated controls or TNF-α-treated cells, in MLN4924-treated cells, binding of NFκB to the MMP9 promoter was inhibited. In the presence of MLN4924, the specific ChIP reaction with an NFκB/p65 antibody yielded results similar to those of the non-specific antibody control (fold enrichment = 1.13 ± 0.66), while use of this antibody in the cells untreated or treated with TNF-α resulted in 1.7 ± 0.04- and 4.5 ± 0.45-fold enrichment of the DNA template, respectively (Figure 4C).

Taken together, these data confirm that MLN4924 treatment results in a loss of interactions between NFκB/p65 and the MMP9 gene promoter.

## 3. Discussion

The migratory ability of tumor cells that allows them to escape the primary sites and to penetrate surrounding and distant tissues is essential for invasion and metastasis [28]. The role of tumor microenvironment (TME)-related signals, including proinflammatory factors, in inducing and promoting cell migration is well-documented and widely accepted [29,30,31]. The proinflammatory cytokine TNF-α is one such factor which is known to enhance cell migration by activation of the NFκB signaling pathway [9,32]. Recently, TNF-α–TNF receptor-2 (TNFR2) signaling has been reported to play an important role in the stimulation of several types of tumors, including ESCC, to become more aggressive [33]. Our previous studies showed that the treatment of breast cancer cells with TNF-α resulted in upregulation of CDKN1A/p21-dependent MMP9 expression/secretion which was found to be essential for cell migration [17,34]. It was consistent with a study on MMP9-deficient mice showing that the loss of MMP9–cell surface interactions dramatically decreases tumor cell motility and invasiveness [35].

In the present study, we observed enhanced cellular migration that was associated with upregulated MMP9 expression and secretion in ESCC cells. Simultaneously, we found that this effect was blocked by MLN4924. In the presence of MLN4924, TNF-α-induced MMP9 expression was totally abrogated while expression of other metalloproteinases, such as MMP2 or MMP14, was not affected at neither the mRNA or protein level. These results are consistent with previous findings of Lan et al. that MLN4924 significantly inhibits gastric cancer cell migration by repressing MMP9 but not MMP2 gene transcription [36].

MLN4924 (pevonedistat) is a small molecule structurally related to adenosine 5′-monophosphate (AMP), with inhibitory activity against the ubiquitin–proteasome system (UPS) which controls concentrations of about 20% of cellular proteins, mainly short-lived signal factors [21,24]. Blocking NAE-dependent neddylation of cullin and consequently CRL-mediated protein ubiquitination by MLN4924 results in the loss of proteostasis due to the accumulation of CRL’s substrates [37]. Among CRL’s substrates there are proteins involved directly or indirectly in the regulation of TNF-α-induced MMP9 gene expression such as IκB-α and AP1/c-Jun or CDKN1A/p21 [17,20,38].

Proteasome-mediated degradation of ubiquitinated IκB-α is essential for the release of NFκB dimers which translocate to the nucleus and bind at the promoter of target genes. Hence, insufficient ubiquitination and consequently inadequate degradation of IκB-α in the presence of MLN4924 significantly impairs activation of NFκB and NFκB-dependent gene expression [24,39,40].

In this study we found that MLN4924 significantly increased levels of phosphorylated IκB-α at Ser32 in a dose- and time-dependent manner. Upregulation of phospho-IκB-α (Ser32) was correlated with a significant decrease in the level of unphosphorylated forms of IκB. This effect characterized the cells treated with MLN4924 alone as well as in combination with TNF-α; however, it seemed independent of TNF-α. In the cells treated with TNF-α alone, phosphorylated forms of IκB-α were degraded within 24 h and the level of the unphosphorylated form only slightly decreased.

Interestingly, MLN4924-dependent upregulation of phospho-IκB-α occurred together with increasing levels of phospho-NFκB/p65 (Ser536) and phospho-NFκB/p65 (Ser529) at unchanged concentrations of the total NFκB/p65 protein. While elevated levels of phosphorylated forms of IκB-α may result from the impairment of proteasome-mediated degradation in the CRL-dependent pathway, the presence of increased phospho-NFκB/p65 levels under MLN4924 is ambiguous. It may be presumed that kinases known to phosphorylate the NFκB/p65 protein, including the IκB kinase (IKK) or casein kinase II (CKII), are influenced by the NEDD8-activating enzyme inhibitor. However, it remains to be elucidated in a future study. It is noteworthy that Noguchi et al. identified NEMO/IKKγ as a non-cullin neddylation substrate. They found that neddylation of NEMO/IKKγ by the RING E3 ligase TRIM40 attenuates NFκB activity and prevents inflammation-associated carcinogenesis in gastrointestinal epithelial cells [41]. The other non-cullin target for neddylation with an effect on NFκB signaling pathway is the BCA3 protein. It was found to have a dose-dependent inhibitory effect on TNF-α-induced NFκB transcriptional activity due to its ability to interact with NFκB/p65 in a neddylation-dependent manner [42]. Hence, inhibition of BCA3 neddylation could enable NFκB/p65 transcription and activation.

Although there is some controversy, it is generally believed that NFκB phosphorylation is an important modification required for the positive regulation of NFκB activity, as well as its nuclear translocation [43,44]. The site-specific phosphorylation of NFκB/p65 controls the stability, degradation and transcription activity of NFκB dimers independently of NFκB-specific inhibitors. However, it is also known to play a major role in the modulation of interactions between NFκB and other factors, including the coactivator CBP (cAMP response element binding (CREB) protein)/p300, to activate chromatin remodeling/transcription complexes in the nucleus [45,46,47].

Although MLN4924 induced phosphorylation of NFκB by itself, it did not lead to translocation and accumulation of NFκB in the nucleus like TNF-α did. However, TNF-α-induced nuclear translocation of the phosphorylated form of NFκB was found to be impaired in the presence of MLN4924. On the other hand, as we observed, the total level of nuclear NFκB in the cells treated with MLN4924 was not different from others. Our coprecipitation (co-IP) assays revealed that nuclear NFκB was able to form complexes with SP1 and AP1/c-Jun in both treated and untreated cells. Interestingly, we found that IκB-α was also coprecipitated with both NFκB and c-Jun proteins from nuclear fractions isolated from all treated and untreated cells. However, contrary to untreated or TNF-α-treated cells, MLN4924-related immune complexes were found to be rich in highly phosphorylated IκB-α and c-Jun and additionally in CDKN1A/p21. It is well-established that the UPS-associated proteolysis allows cells to regulate precisely most of the intracellular processes in the cytoplasm and also in the cell nucleus. Disruption of the proper balance in the proteasomal degradation can contribute to the formation of nonfunctional complexes or clusters of inappropriate proteins leading to an inadequate response to extracellular stimuli [48]. As previously indicated, AP1/c-Jun and CDKN1A/p21 are proteins that are primarily degraded through the ubiquitin–proteasome pathway and abnormally accumulated in cells under the treatment with MLN4924 [38,49]. We found that MLN4924 also caused a time- and dose-dependent increase in levels of these two proteins in ESCC cells. CDKN1A/p21, one of the cyclin-dependent kinase (Cdk) inhibitors, is well-known as a critical mediator of cell cycle arrest, an inducer of cell senescence and a tumor suppressor. However, in addition to these roles, CDKN1A/p21 was recently found to promote cell proliferation and migration [49]. In our previous study, we found that CDKN1A/p21 was upregulated under TNF-α and served as a positive regulator of TNF-α-induced MMP9 gene expression in breast cancer cells [17]. The relationship between MMP9 expression/activity and CDKN1A/p21 expression in response to TNF-α treatment was found to also be characteristic of ESCC cells as the present study showed. However, increasing levels of CDKN1A/p21 in the presence of MLN4924 did not correlate with MMP9 expression and activity.

In this study, we also observed that increasing levels of total c-Jun positively correlated with c-Jun phosphorylation at Ser73. The c-Jun protein is an AP-1 family member of dimeric transcription factors. Its functional role may vary depending on the cell type and dimer composition. However, it is generally thought to have an oncogenic effect through being involved in the regulation of a wide range of genes associated with hallmarks of cancers, including upregulation of MMP9 [50,51].

It is well-known that effective gene expression requires precisely coordinated interactions of multiple transcription factors. Phosphorylation/dephosphorylation events are considered among the most important modulators of their activity [52]. For instance, phosphorylation at Ser63 and Ser73 increases the DNA-binding capacity of c-Jun. Nevertheless, heavy phosphorylation was shown to lower the transactivation potential of c-Jun [53]. Since NFκB/p65-c-Jun-IκB-α-SP1 complexes were formed in all the cells, both untreated controls and TNF-α- or MLN4924-treated cells, we assume that the NFκB/p65 transcriptional activation in the presence of MLN4924 was blocked by the overload of nuclear NFκB/p65 complexes with highly phosphorylated IκB and AP1/c-Jun forms which prevent NFκB/p65:DNA interaction. Our results indicate that MLN4924 may impair not only the cytoplasmic, but also the nuclear UPS activity. In recent years, it has become evident that the ubiquitin-mediated proteasomal degradation system is involved in gene control [47,54]. The nuclear UPS was found to regulate the turnover of many different components of transcriptional complexes [54]. Particular attention was paid to the role of cullin 1 (CUL1) and cullin 4B (CUL4B) [55]. On the other hand, it is known that in addition to the inhibitory effects of neddylation, MLN4924 also shows neddylation-independent activities. Zhao et al. observed that knockdown of NEDD8 did not mimic MLN4924 in activating JNK signaling in human head and neck squamous cell carcinoma (HNSCC) cell lines [56]. MLN4924 was found to trigger epidermal growth factor receptor (EGFR) dimerization and activate EGFR downstream signaling pathways such as RAS/RAF/MEK/ERK and PI3K/AKT1/mTOR promoting tumor sphere formation and inducing wound healing in mouse models. It was also able to initiate PKM2 tetramerization and stimulate glycolysis [57]. These pro-survival neddylation-independent activities of MLN4924 may indicate that MLN4924 can also serve as a stimulator of certain cells.

## 4. Materials and Methods

### 4.1. Cell Culture and Treatment

Human esophageal squamous cell carcinoma cell lines KYSE70 and KYSE150 were purchased from Merck, Kenilworth, NJ, USA (European Collection of Authenticated Cell Cultures, ECACC 94072012) and Leibniz-Institut DSMZ-German Collection of Microorganisms and Cell Cultures, Braunschweig, Germany (ACC 375), respectively. The KYSE70 cells were grown in the RPMI 1640 medium (Biowest, Riverside, MO, USA, L0501-500) supplemented with 10% FBS South America, Heat Inactivated (Biowest, S181H-500) and 2 mM l-glutamine (Biowest, X0550-100). The KYSE150 cell line was cultured in the RPMI 1640 medium supplemented with 2% FBS and 2 mM l-glutamine. The cultures were maintained at 37 °C in a humidified 5% CO_2_/95% air atmosphere.

Cells at 70% confluency were starved overnight in a serum-free medium and then treated with MLN4924 (pevonedistat) (Axon Medchem, Groningen, The Netherlands, 2038) at the concentration of 1 µM and/or TNF-α (30 ng/mL) (Prospec, Ness-Ziona, Israel, cyt-223-b) for 24 h, unless otherwise specified. The conditioned media were collected, suspended in 2× non-reducing sample buffer (62.5 mM Tris-HCl, pH 6.8, with 10% glycerol, 2% SDS, 0.05% bromophenol blue) and used immediately in gelatin zymography. The cells were lysed (5 × 10^6^ cells/mL) with 5× Passive Lysis Buffer (Promega, Madison, WI, USA, E1941) supplemented with a protease and phosphatase inhibitor cocktail (Cell Signaling, Danvers, MA, USA, 5872). Cell lysates were centrifuged at 16,000× *g* for 15 min at 4 °C. The supernatants were collected, and the protein concentration was determined using a Precision Red Advanced Protein Assay (Cytoskeleton, Denver, CO, USA, ADV02-A). Samples were reduced with 4× Laemmli sample buffer (Bio-Rad, Hercules, CA, USA, 1610747) at 95 °C for 10 min and stored at −20 °C until use. Effects of MLN4924 and TNF-α on cell viability were determined using Trevigen’s TACS MTT Cell Proliferation Assay (R&D Systems, Minneapolis, MN, USA, 4890-050-K) according to the manufacturer’s instructions.

### 4.2. Gelatin Zymography

To test MMP9 and MMP2 activity, samples were electrophoresed without boiling in 10% native polyacrylamide gel containing 2 mg/mL gelatin. The gels were washed twice with a 50 mM Tris-HCl (pH 7.5) buffer supplemented with 2.5% Triton X-100 and incubated overnight at 37 °C in a 50 mM Tris-HCl (pH 7.5) buffer containing 150 mM NaCl, 10 mM CaCl_2_, 1 μM ZnCl_2_ and 0.05% Brij-35. Gels were stained with 0.125% (*w*/*v*) Coomassie Brilliant Blue R-250, 62.5% (*v*/*v*) methanol and 25% (*v*/*v*) acetic acid solution. Gelatinolytic activity was visualized as unstained bands on a dark blue background.

### 4.3. Cell Fractionation

To obtain the cytoplasmic extract, cells (8.5 × 10^6^ per extract) were washed with PBS, resuspended in 100 μL of a 10 mM HEPES (4-(2-hydroxyethyl)-1-piperazineethanesulfonic acid, pH 7.6) buffer containing 60 mM KCl, 1 mM ethylenediaminetetraacetic acid (EDTA), 0.075% (*v*/*v*) NP-40, 1 mM dithiothreitol (DTT) and 1 mM phenylmethylsulfonyl fluoride (PMSF) and centrifuged at 400× *g* for 4 min at 4 °C. To obtain the nuclear extract, the pellet remaining after centrifugation was washed with a 100 μL 10 mM HEPES (pH 7.6) buffer without detergent and resuspended in 100 μL of a lysis buffer (420 mM NaCl, 1.5 mM MgCl_2_, 0.2 mM EDTA, 1 mM PMSF and 25% (*v*/*v*) glycerol, 20 mM tris(hydroksymethyl)aminomethane (Tris)-HCl, pH 8.0) supplemented with the protease and phosphatase inhibitors. Samples were cleared by centrifugation at 16,000× *g* for 10 min at 4 °C.

### 4.4. Coimmunoprecipitation and Western Blotting

For coimmunoprecipitation (co-IP) of the protein complexes, the nuclear extracts (500 µg/mL) were mixed with rabbit or mouse antibodies directed against NFκB/p65 from Abcam (ab16502, Cambridge, UK) and Cell Signaling (6956), respectively, and incubated on a rocking platform overnight at 4 °C. The incubation mixtures were then mixed with 35 μL of Protein G magnetic beads (Invitrogen, Waltham, MA, USA, 10003D) and incubated for the next 2 h at 4 °C. The beads were washed three times with a 10 mM HEPES (pH 7.6) buffer, and complexes of proteins bound to the antibody-anchored beads were released by boiling in 2× Laemmli sample buffer (Bio-Rad, 1610747) at 95 °C for 10 min.

For the Western blot analysis, the protein samples were separated by SDS-PAGE and transferred to 0.2 μm nitrocellulose membranes (Bio-Rad, 1620112). Membranes were blocked with 5% nonfat milk in PBS, followed by overnight incubation at 4 °C with an appropriate primary antibody diluted in PBS. Rabbit anti-β-Actin (13E5) (4970), c-Jun (60A8) (9165), Phospho-c-Jun (Ser73) (D47G9) XP^®^ (3270), Phospho-IκBα (Ser32) (14D4) (2859), Phospho-NF-κB p65 (Ser536) (3033), p21 Waf1/Cip1 (12D1) (2947), SP1 (D4C3) (9389S), mouse anti-IκBα (L35A5) (4814) and NF-κB p65 (L8F6) (6956) antibodies were products of Cell Signaling. Rabbit anti-NFκB p65 (ab16502) antibody was acquired from Abcam, and anti-MMP14 (PA5-13183) was purchased from Thermo Fisher Scientific, Waltham, MA, USA. Mouse anti-PARP-1 Antibody (C2-10) was obtained from Santa Cruz Biotechnology, Dallas, TX, USA (sc-53643). After three washes with 0.1% Tween-20 in PBS (PBS-T), membranes were incubated with the appropriate horseradish peroxidase-conjugated secondary antibody or with protein A conjugated with HRP purchased from Merck Millipore, Burlington, MA, USA (18-160). Rabbit anti-mouse IgG (A16166) was a product of Invitrogen, and goat anti-rabbit IgG (7074) was obtained from Cell Signaling. Immunocomplexes were visualized by chemiluminescence in a 100 mM Tris buffer, pH 8.5, supplemented with p-coumaric acid (Sigma-Aldrich, St. Louis, MO, USA, 9008), luminol (Sigma-Aldrich A891) and H_2_O_2_ using an Azure 280 detector. After stripping with a mild stripping buffer (0.2 M glycine, pH 2.2, 10% Tween 20, 1% SDS), the membranes were re-probed with other antibodies.

### 4.5. Profiling NFκB Signaling Pathway Proteins

To determine the relative levels of 41 total and 4 serine/tyrosine phosphorylated proteins involved in NFκB signal transduction, the Proteome Profiler Human NF-κB Pathway Array (R&D Systems, ARY029) was used. KYSE150 cells after 24-h starvation were treated with MLN4924 and TNF-α alone or in combination for 1 h and then processed according to the manufacturer’s protocol. Immunostained dots were visualized using the Azure 280 detector. Pixel densities were analyzed using ImageJ software (https://imagej.nih.gov/ij/, accessed on 28 January 2021).

### 4.6. Immunofluorescence (IF) Staining

The cells were seeded on round coverslips (Ø 10 mm) and after treatment they were washed with PBS, fixed with 4% paraformaldehyde for 10 min, permeabilized with PBS containing 0.1% Triton X-100 and incubated with a blocking buffer (4% horse serum in PBS). The cells were then subjected to immunofluorescence staining with a rabbit anti-Phospho-NFκB p65 (Ser536) antibody (Cell Signaling, 3033) (diluted 1:100 in a blocking buffer) overnight at 4 °C. After that, the cells were washed with PBS and incubated with Alexa 568-labeled anti-rabbit secondary antibody (diluted 1:1000 in a blocking buffer) (Invitrogen, A-11011) at room temperature for 1 h. Slides were mounted using ProLong Gold antifade with a DAPI reagent (Invitrogen, P36931). Digital images were acquired using a Carl Zeiss Axio Vert.A1 inverted microscope (ZEISS, Oberkochen, Germany) with an Ec Plan Neofluar 10× 0.3 Ph1 objective.

### 4.7. Chromatin Immunoprecipitation (ChIP) Assay

Chromatin immunoprecipitation of NFκB binding with the promoter of the MMP9 gene was performed using chromatin prepared from 10^7^ KYSE150 cells treated with MLN4924 (1 µM) or TNF-α (30 ng/mL) for 24 h and 1 h, respectively. The chromatin was fixed using formaldehyde in PBS at a final concentration of 1% for 10 min. After neutralization with 2 M glycine, pH 7.5, for 5 min, the cells were washed and scraped into 1 mL of PBS, centrifuged at 400× *g* for 5 min and lysed in 1 mL of a swelling buffer (25 mM HEPES, pH 7.8, 1.5 mM MgCl, 10 mM KCl, 0.1% Igepal, 1 mM DTT) and then centrifuged at 2500× *g* for 10 min. Nuclei pellets were resuspended in 100 µL of a sonication buffer (150 mM Tris-HCl, pH 8.1, 500 mM NaCl, 1 mM EDTA, 1% TritonX-100, 0.1% Na-deoxycholate and 1% SDS). The chromatin was sheared by sonication using an ultrasonic processor UP100H (Hielscher Ultrasonics, Teltow, Germany) in 6 cycles of 30 s ON/OFF with 40% amplitude. The samples were centrifuged at 14,000× *g* for 10 min and supernatants containing sheared chromatin were diluted with 10× ChIP dilution buffer (16.7 mM Tris-HCl, pH 8.1, 16.7 mM NaCl, 1.2 mM EDTA, 1.1% TritonX-100, 0.1% sodium deoxycholate and 0.01% SDS) and used for immunoprecipitation with 4 μg of a rabbit antibody directed against NFκB (ab16502, Abcam), and for input (2% volume of the supernatant). For appropriate controls, the ChIPAb + Histone H3.3 set (17-10245, Millipore), including the Histone H3.3 antibody, a normal rabbit IgG and control primers, was used. Immune complexes were collected using ChIP-grade protein A/G magnetic beads (Thermo Fisher Scientific, 26162) and washed with low- (20 mM Tris-HCl, pH 8.1, 150 mM NaCl, 2 mM EDTA, 1% TritonX-100, 0.1% SDS) and high-salt (20 mM Tris-HCl, pH 8.1, 500 mM NaCl, 2 mM EDTA, 1% TritonX-100, 0.1% SDS) buffers and then with a LiCl wash buffer (100 mM Tris-HCl, pH 9.0, 0.25 mM LiCl, 1 mM EDTA, 1% Igepal, 0.1% sodium deoxycholate). Protein–DNA complexes were eluted with an elution buffer (0.1 M NaHCO_3_, pH 8.0, containing 1% SDS), heated at 95 °C for 15 min and after cooling digested with ribonuclease A (Thermo Fisher Scientific, EN0531) and proteinase K (Ambion, Austin, TX, USA, AM2546) for 2 h at 60 °C. Fragments of DNA were purified with a QIAquick PCR Purification Kit (Qiagen, Hilden, Germany, 28104) according to the manufacturer’s instructions and used as a template for real-time PCR amplification using primers covering (as positive controls) the MMP9 promoter (from −1898 to −1764): forward, 5′-T CGT GAC TGC AAA GCA GAT GTT-3′ and reverse, 5′-G CCT ACT ATG TGC CAG GCA TTT TA-3′; and the GAPDH promoter: forward, 5′-GCC ATG TAG ACC CCT TGA AGA G-3′ and reverse, 5′-ACT GGT TGA GCA CAG GGT ACT TTA T-3′. The PCR products (134 bp and 87 bp for MMP9 and GAPDH, respectively) were resolved by 2% agarose gel electrophoresis and visualized by ethidium bromide staining.

### 4.8. Quantitative Polymerase Chain Reaction (qPCR)

Total RNA was isolated from treated or untreated cells using the RNeasy Mini Kit (Qiagen, 74104) according to the manufacturer’s instructions. cDNA was synthesized from 2 μg of total RNA using the SuperScript IV VILO Master Mix with ezDNase Enzyme (Invitrogen, 11766050). Real-time PCR was performed with SYBR Green and Mic qPCR Cycler (Bio Molecular Systems, Upper Coomera, QLD, Australia). Five microliters of 2× SsoFast EvaGreen Supermix (Bio-Rad, 172-5200) were mixed with 0.2–1 μL of final cDNA volume and forward and reverse primers (10 μM) in a final reaction volume of 10 μL. Cycling conditions were 30 s at 95 °C, 40 cycles of 10 s at 95 °C and 15 s at 60 °C or 64 °C. Each sample was run in triplicate. Amplification efficiency was assessed for all primer sets used in separate reactions, and primers with efficiencies in the range of 95–110% were used. Primers for the CDKN1A gene were purchased from Sino Biological, Beijing, China (HG11108-ACG). Primers for MMP9 were products of OriGene, Rockville, MD, USA (HP208217). To normalize gene expression, primers for glyceraldehyde 3-phosphate dehydrogenase (GAPDH) (OriGene, HP100003) were used. The length of generated products was 94 bp, 114 bp and 108 bp, respectively. The ΔCt was calculated by subtracting the cycle threshold (Ct) value of GAPDH mRNA from the Ct value of the gene of interest (ΔCt). Fold change in the gene expression was calculated using the 2^−ΔΔCt^ method.

### 4.9. Wound Healing Assay

KYSE70 cells were plated on 12-well plates and grown to 75–80% confluence in a complete medium and then they were serum-starved for 24 h. The cell-free gaps were created by an ibidi Culture-Insert 3 Well (80369). The cells were then treated with 1 µM MLN4924 alone or in combination with 30 ng/mL of TNF-α in a serum-free medium. The pictures (2464 × 2056 pix) were captured at 24 and 48 h at 20× magnification using a Zeiss digital camera integrated with an Axio Vert.A1 inverted microscope (Carl Zeiss). The area of the wound was quantified using ImageJ software. The cell migration was expressed as the ratio of wound closure (R): R = ((A _0 h_ − A _∆ h_)/ A _0 h_), where A _0 h_ is the area of the cell-free gap measured immediately after the insert was removed and A _∆ h_ is the area of the artificial wound measured after 24 or 48 h.

### 4.10. Statistical Analysis

If not otherwise specified, data from at least three independent experiments were analyzed using an independent *t*-test (Statistica 13). The *p*-value ≤ 0.05 was considered to indicate a statistically significant difference.

## 5. Conclusions

We first revealed that an NEDD8-activating enzyme inhibitor exhibits an inhibitory effect on the TNF-α-induced activity of MMP9 in ESCC cells and therefore suppresses MMP9-dependent cancer cell migration. Hence, this report was aimed at providing more detailed information on a mechanism by which MLN4924 prevents MMP9 expression. We found that in the presence of MLN4924 interactions between NFκB and the MMP9 gene promoter are totally blocked. Disruption of the stoichiometric balance among components of the NFκB complex, likely through MLN4924-dependent disturbances in the nuclear ubiquitin–proteosome system, appeared to be the main culprit in this case. We identified three CRL substrates, IκB, c-Jun and CDKN1A/p21, as potentially responsible for the loss of functionality of the NFκB complex in the nucleus. Our results emphasize the important role of nuclear UPS in the regulation of transcriptional processes, in particular when it comes to anticancer therapy.

## Supplementary Materials

The following are available online at https://www.mdpi.com/1422-0067/22/4/1716/s1, Figure S1: Effect of MLN4924 pretreatment on CDKN1A/p21 and c-Jun protein level in cells treated with TNF-α in different concentrations; Figure S2: Effect of MLN4924 and TNF-α on cell viability in KYSE70 and KYSE150 cells.; Figure S3: PARP1 cleavage in response to MLN4924 in ESCC cells. KYSE150 cells were treated with various concentrations of MLN4924 alone or in combination with TNF-α (30 ng/mL) for 24 h.; Figure S4: Analysis of complete proteome profiler NFκB pathway of KYSE150 cells.; Figure S5: A dose-dependent effect of MLN4924 on the signaling pathways mediating MMP9 gene expression in KYSE70 cells.
