# Supplementary material for: The Effect of Neddylation Inhibition on Inflammation-Induced MMP9 Gene Expression in Esophageal Squamous Cell Carcinoma
Source: Int J Mol Sci. 2021 Feb 9;22(4):1716. doi: 10.3390/ijms22041716 (PMC7915196; doi:10.3390/ijms22041716)
Supplement: Supplementary file 1 [file ijms-22-01716-s001.pdf]

International Journal of *Molecular Sciences*

MDPI

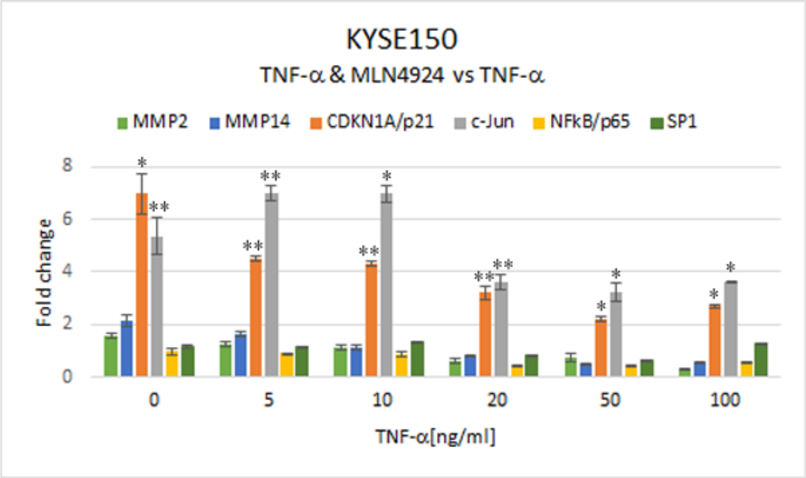

Figure S1. Effect of MLN4924 pretreatment on CDKN1A/p21 and c-Jun protein level in cells treated with TNF-α in different concentrations. The bar graph presents data as relative fold change in *MMP2, MMP14, CDKN1A, c-Jun, NFκB* and *SP1* protein levels at the *TNF-α* concentration range from the mean value of MLN4924 (1 μM) pretreated cells divided by the mean value of non-pre-treated cells, quantified by densitometry analysis and normalized to housekeeping ACTB protein (B). Values shown are means ± SEM. * $p < 0.05$, ** $p < 0.01$.

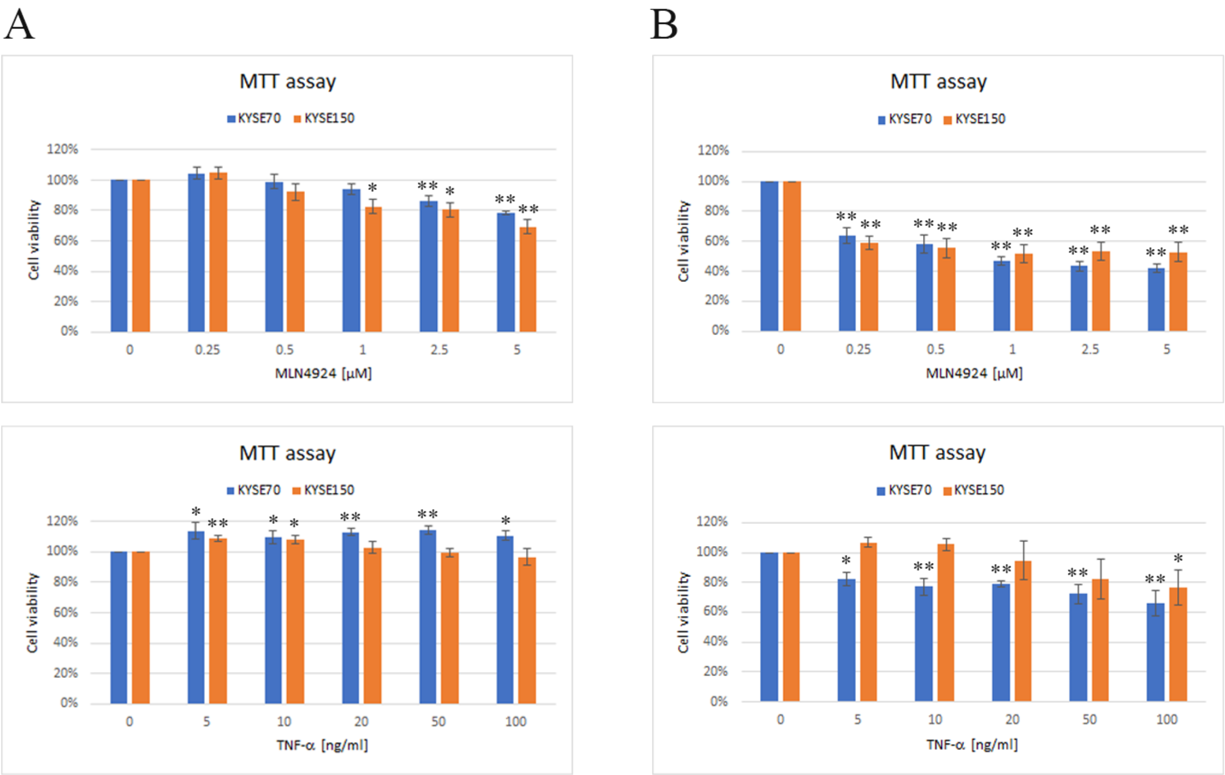

Figure S2. Effect of MLN4924 and TNF-α on cell viability in KYSE70 and KYSE150 cells. ESCC cells were treated with various concentrations of MLN4924 (0 to 5 μM) and TNF-α (0–100 ng/mL) for 24 (**A**) and 48 h (**B**). Viability of cells was determined by MTT assay. Data are presented as the mean of two experiments with four replicates each ± SEM and are expressed as the percentage of untreated controls. * $p < 0.05$, ** $p < 0.001$ represents a significant difference compared to controls.

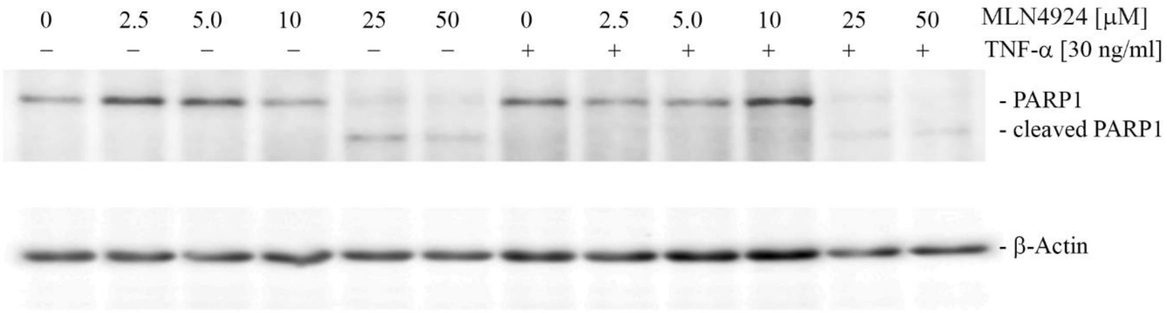

Figure S3. PARP1 cleavage in response to MLN4924 in ESCC cells. KYSE150 cells were treated with various concentrations of *MLN4924* alone or in combination with TNF-α (30 ng/mL) for 24 h. The full-length PARP1 and apoptotic fragments (89 kDa) were analyzed by Western blotting. ACTB was used as a loading control.

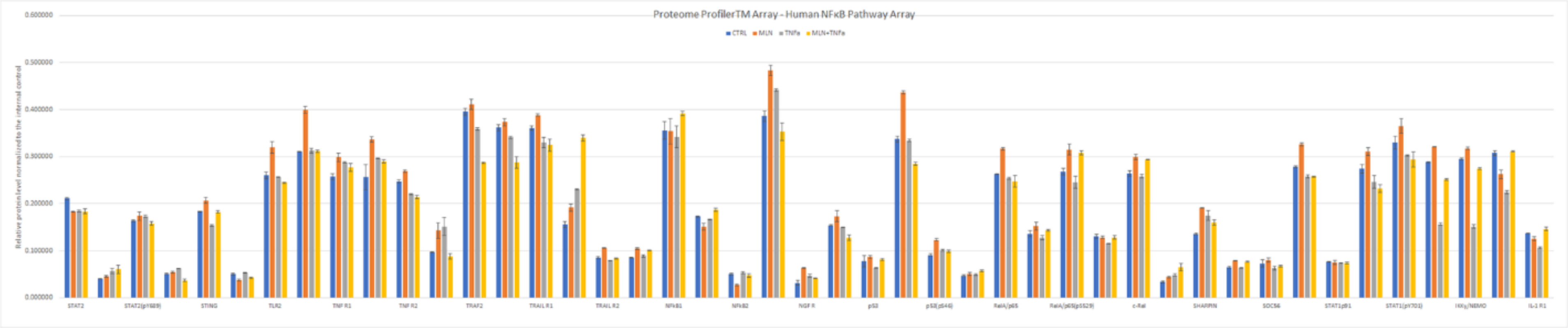

Figure S4. Analysis of complete proteome profiler NFκB pathway of KYSE150 cells. Bar graphs showing semi-quantitative analysis of selected proteins. KYSE150 cells were untreated or treated with 30 ng/mL TNF-α for 1 h with or without pretreatment with 1 μM MLN4924 for 24 h. Results are presented as means ± SEM from duplicates.

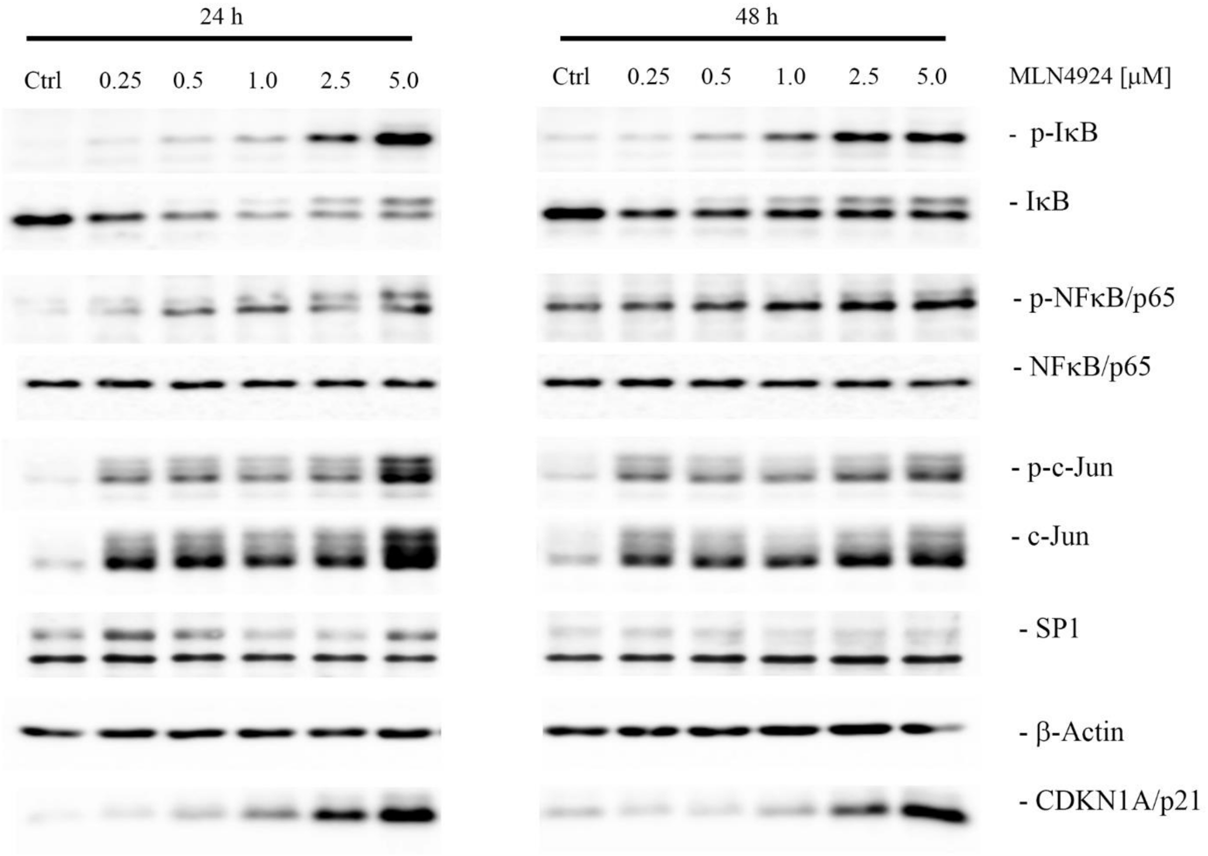

Figure S5. A dose-dependent effect of MLN4924 on the signaling pathways mediating *MMP9* gene expression in KYSE70 cells. Western blot analysis showing changes in activation of IκB-α, NFκB/p65 and c-Jun as well as increasing levels of CDKN1a/p21 protein in KYSE70 cells under the treatment with different concentrations (0.25, 0.5, 1.0, 2.5 and 5.0 μM) of MLN4924 for 24 and 48 h. ACTB was used as a loading control.
